# Novel Finding of Coronary Ectasia in a Case of Acute Rheumatic Fever

**DOI:** 10.1155/2013/674174

**Published:** 2013-07-09

**Authors:** Thomas Weiler, Anjali Chelliah, Linda Bradley-Tiernan, E. Anne Greene

**Affiliations:** ^1^Pediatric Residency Program, Children's National Medical Center, 111 Michigan Avenue, NW, Washington, DC 20010, USA; ^2^Division of Cardiology, Children's National Medical Center, 111 Michigan Avenue, NW, Washington, DC 20010, USA

## Abstract

A 10-year-old boy presented to his pediatrician with acute fever, rash, and polyarthritis. Laboratory studies revealed elevated inflammatory markers and positive throat culture. Echocardiogram demonstrated panvalvular insufficiency consistent with acute rheumatic fever (ARF) and coronary artery ectasia. This latter finding, typically associated with Kawasaki disease, has not been previously reported in ARF.

## 1. Introduction

 Acute rheumatic fever (ARF) results from a cellular and humoral autoimmune response after infection by *Streptococcus pyogenes* [1]. Clinically diagnosed using the Jones criteria (Table 1) [2, 3], it typically occurs about three weeks after group A streptococcus infection. Carditis involving the valves, myocardium, and/or pericardium occurs in 30–70% of patients with ARF [4].

The current literature provides examples of coronary vasculitis in the acute phase of ARF as well as more indolent inflammation associated with rheumatic heart disease [5], but no cases of coronary ectasia have been documented in ARF. Our report highlights this unusual finding and the importance of accurate diagnosis and long-term treatment.

## 2. Patient Presentation

A 10-year-old previously healthy boy presented to his pediatrician with a four-day history of sore throat and daily fever. He also reported two days of erythematous rash over his extremities and mild abdominal pain. Rapid strep antigen test was negative, and his family was advised to continue supportive care. When throat culture became positive for group A streptococcus, he was started on amoxicillin, but after one day of antibiotics he developed pain, erythema, and edema in his right ankle, knee, and subsequently in his left elbow. The family discontinued treatment due to concern for drug reaction and returned to their pediatrician. Since the onset of fever, he reported no conjunctivitis, mucous membrane changes, or swelling of his hands or feet.

Laboratory studies two days after throat culture included a CBC (WBC 14,000/*μ*L, hemoglobin 10.7 gm/dL, and platelets 368,000/*μ*L) and an ESR elevated at 115 mm/hour (normal: 0–15). ANA was negative. At this time, he was referred to a pediatric cardiologist for evaluation for ARF.

In the cardiology clinic, the patient was found to be ill-appearing with diffuse joint pain. Temperature was 38.4°C, HR 65, RR 15, and BP 112/53. Skin was unremarkable with resolution of previous rash. Lungs were clear. On cardiac exam, he had normal precordial activity and PMI. Rhythm was regular with normal first and second heart sounds. A grade 2/6 regurgitant murmur was heard at the left sternal border. Liver edge was palpable at the right costal margin, and pulses were equal with no femoral delay. No conjunctivitis, lymphadenopathy, mucous membrane changes, hand/foot swelling, or joint erythema or edema were present.

**Figure 1:**
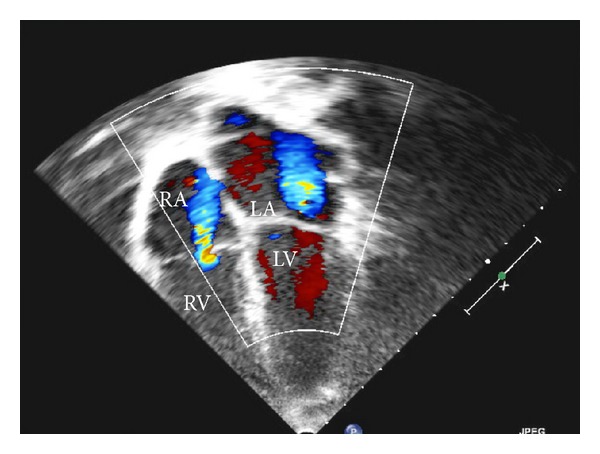
Apical 4-chamber view showing mitral valve (MV) and tricuspid valve (TV) regurgitations (demonstrated by the blue jets of retrograde flow on the Doppler study). Aortic and pulmonary valve regurgitations are not seen on this image.

**Figure 2:**
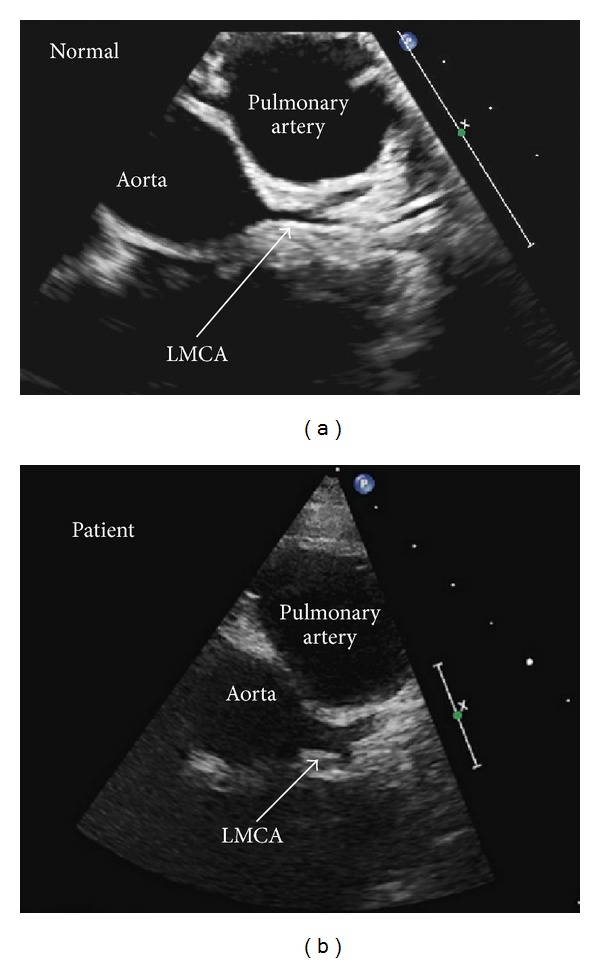
Parasternal short axis view showing dilated LMCA of our patient with normal shown for comparison.

Twelve-lead electrocardiogram showed an accelerated junctional rhythm at 67 bpm and borderline QTc prolongation (450 msec). Echocardiogram showed normal systolic and diastolic function, but Doppler demonstrated mild insufficiency of the aortic, pulmonary, mitral, and tricuspid valves (Figure 1). The left main coronary artery (LMCA) was diffusely enlarged (4.8 mm, Z-score +2.9) (Figure 2) (See video 1 in the Supplementary Material available online at http://dx.doi.org/10.1155/2013/674174). The left anterior descending (LAD) (2.4 mm, Z-score −0.08) and right coronary (3.1 mm, Z-score +1.0) arteries were normal.

Given patient's history of fever, streptococcal pharyngitis, rash, migratory polyarthritis, elevated inflammatory markers, and carditis, he was admitted to our pediatric tertiary care center for treatment of ARF.

## 3. Hospital Course

**Figure 3:**
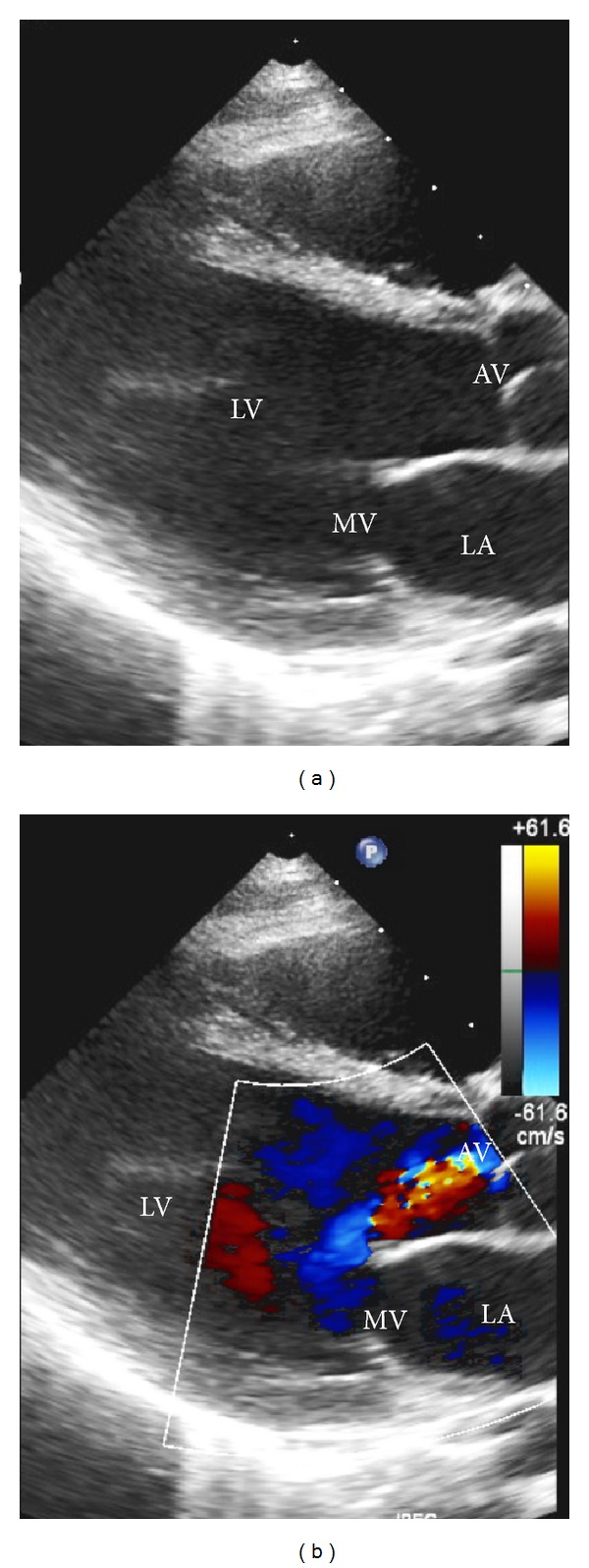
Parasternal long axis view showing (a) orientation of the aortic valve (AV), left ventricle (LV), and left atrium (LA) and (b) aortic valve (AV) and mitral valve (MV) regurgitations demonstrated by blue jets of retrograde flow on Doppler.

Treatment was started with ceftriaxone for ARF and aspirin for management of his fever and joint pain [1]. Laboratory studies demonstrated an antistreptolysin O titer of 1458 IU/mL (normal <150) and anti-DNAse B of 533 U/mL (normal <376). On hospital day 3, echocardiogram showed stable pulmonic, tricuspid, and mitral insufficiency but mildly worsening aortic insufficiency and enlargement of the LMCA (5.4 mm, Z-score +3.6). The worsening coronary ectasia with prolonged fever raised concern for possible atypical Kawasaki disease (KD), and the patient was treated with a dose of intravenous immunoglobulin. He was also started on prednisolone for persistent carditis in the setting of ARF [6]. Echocardiogram the next day demonstrated stable LMCA dilation but further progression of aortic insufficiency (Figure 3) (video 2) and new dilation of the LAD (4.0 mm, Z-score +2.7). With these findings, in consultation with the rheumatology team, prednisolone was changed to a three day course of methylprednisolone [7]. Echocardiogram on hospital day 8 showed improved LMCA (4.2 mm, Z-score +2.1) and LAD (3.4 mm, Z-score +1.9) dilations, with stable valvular insufficiency.

Upon discharge, the patient completed antibiotic treatment for ARF with plans to continue penicillin prophylaxis. He finished a total of six weeks of high-dose aspirin and two more weeks of prednisolone, each with a subsequent taper. 

At cardiology followup two weeks after discharge, he had no rash or joint findings. Exam showed a grade 2/6 regurgitant murmur at the apex and a persistent grade 1/6 regurgitant murmur at the left midsternal border. Echocardiography demonstrated significant improvement in insufficiency of all valves, stable LMCA ectasia (4.5 mm, Z-score +2.4), and resolution of LAD ectasia (3.0 mm, Z-score +0.96). At a subsequent followup echocardiogram one month later, patient's LMCA ectasia showed regression towards normal (3.8 mm, Z-score +1.3).

## 4. Discussion

 In children, coronary ectasia is typically associated with KD. This syndrome is characterized by global vascular inflammation associated with coronary ectasia caused by antiendothelium antibodies. Inflamed vascular walls succumb to hemodynamic stress, leading to coronary dilation or aneurysms [3]. 

This case is the first to describe coronary ectasia in a patient who met diagnostic criteria for ARF. While coronary ectasia can exist in other acute febrile illnesses, Z-scores ≥2.5, as seen in this patient, have been considered specific for KD [8] and have been used to confirm the diagnosis in cases with incomplete clinical criteria [9].

Given the very different treatment and prognostic implications of ARF and KD, it becomes extremely important to correctly distinguish between the two in the presence of a finding such as coronary ectasia. ARF is diagnosed using the Jones criteria [2] and evidence of a preceding streptococcal infection. KD diagnosis typically requires five days of fever and four out of five relevant clinical criteria [3] (Table 1). Treatment of KD with transient coronary ectasia after the acute phase consists of four to six weeks of aspirin therapy [3], whereas long-term care of pediatric patients with ARF includes antibiotic prophylaxis until 21 years of age or 10 years after the last episode of ARF [10]. One large series showed that 65% of ARF patients not treated with antibiotic prophylaxis went on to develop valvular heart disease with an overall annual mortality rate of 1.5% [11]. Given their vastly different therapies, misdiagnosis of ARF with coronary ectasia as KD could place a patient at risk of the long-term sequelae of rheumatic heart disease.

This case broadens the known spectrum of cardiac involvement in ARF and emphasizes the need for to consider both ARF and KD in the differential diagnosis of coronary artery ectasia. It is possible that the cardiac sequelae of these two diseases may be more similar than previously thought and may represent different manifestations of a single inflammatory cascade with unrecognized triggers and differentiating mechanisms. Future research may explore the overlap in these pathophysiologic mechanisms and will likely help understand both diseases.

## Supplementary Material

Video 1: The initial echocardiogram of the patient in this case shows a parasternal short axis view demonstrating flow through the dilated LMCA.
Video 2: Subsequent echocardiogram shows a parasternal long axis view. This video demonstrates progression of aortic valve (AV) and mitral valve (MV) regurgitation from previous images.

## Figures and Tables

**Table 1:** Clinical manifestations of acute rheumatic fever versus Kawasaki disease found in the reported case (noted with *√* symbol).

Acute rheumatic fever (major Jones criteria) [2]	Kawasaki disease (clinical diagnostic criteria) [3]
Migratory arthritis (usually large joints)^*√*^	Bilateral bulbar conjunctival injection
Carditis and valvulitis^*√*^	Oral mucous membrane changes
Central nervous system involvement (chorea)	Peripheral extremity changes
Erythema marginatum^*√*^	Polymorphous rash^*√*^
Subcutaneous nodules	Cervical lymphadenopathy

## Abbreviations

ANA: Antinuclear antibody
ARF: Acute rheumatic fever
AV: Aortic valve
ESR: Erythrocyte sedimentation rate
KD: Kawasaki disease
LA: Left atrium
LAD: Left anterior descending coronary artery
LMCA: Left main coronary artery
LV: Left ventricle
MV: Mitral valve
QTc: Corrected QT interval
TV: Tricuspid valve.
